# Exploring Novel Variants of the Cytochrome P450 Reductase Gene (*POR*) from the Genome Aggregation Database by Integrating Bioinformatic Tools and Functional Assays

**DOI:** 10.3390/biom13121728

**Published:** 2023-11-30

**Authors:** Maria Natalia Rojas Velazquez, Søren Therkelsen, Amit V. Pandey

**Affiliations:** 1 Division of Pediatric Endocrinology, Department of Pediatrics, University Children’s Hospital Bern, 3010 Bern, Switzerland; maria.rojasvelazquez@students.unibe.ch (M.N.R.V.); fsx728@sund.ku.dk (S.T.); 2 Translational Hormone Research, Department of Biomedical Research, University of Bern, 3010 Bern, Switzerland; 3 Graduate School for Cellular and Biomedical Sciences, University of Bern, 3010 Bern, Switzerland; 4 Department of Drug Design and Pharmacology, University of Copenhagen, 1172 Copenhagen, Denmark

**Keywords:** cytochrome P450 oxidoreductase, POR, gnomAD, CAH, drug metabolism, steroid hormones, SNV, bioinformatics

## Abstract

Cytochrome P450 oxidoreductase (POR) is an essential redox partner for steroid and drug-metabolizing cytochromes P450 located in the endoplasmic reticulum. Mutations in *POR* lead to metabolic disorders, including congenital adrenal hyperplasia, and affect the metabolism of steroids, drugs, and xenobiotics. In this study, we examined approximately 450 missense variants of the *POR* gene listed in the Genome Aggregation Database (gnomAD) using eleven different in silico prediction tools. We found that 64 novel variants were consistently predicted to be disease-causing by most tools. To validate our findings, we conducted a population analysis and selected two variations in *POR* for further investigation. The human POR wild type and the R268W and L577P variants were expressed in bacteria and subjected to enzyme kinetic assays using a model substrate. We also examined the activities of several cytochrome P450 proteins in the presence of POR (WT or variants) by combining P450 and reductase proteins in liposomes. We observed a decrease in enzymatic activities (ranging from 35% to 85%) of key drug-metabolizing enzymes, supported by POR variants R288W and L577P compared to WT-POR. These results validate our approach of curating a vast amount of data from genome projects and provide an updated and reliable reference for diagnosing POR deficiency.

## 1. Introduction

POR plays a fundamental role as the primary electron donor for all type II cytochrome P450 enzymes located in the endoplasmic reticulum [1,2,3,4] and is a central part of the microsomal oxidase system [5,6]. These P450 enzymes include CP21A2, CYP17A1, and CYP19A1 responsible for steroid biosynthesis, as well as CYP51A1 and CYP26B1 contributing to bone formation [7,8,9]. Homozygous or compound heterozygous mutations in the human *POR* gene (MIM: *124015) give rise to a diverse spectrum of clinical manifestations known as *POR* deficiency (PORD; MIM: #613571) in an autosomal recessive inheritance [7,10,11,12,13,14,15,16,17,18]. The severest forms of PORD encompass congenital adrenal hyperplasia (CAH) [18,19,20,21], disruptions in sexual and pubertal development (DSD), and skeletal anomalies resembling Antley–Bixler syndrome with genital anomalies and disordered steroidogenesis (ABS1; MIM: #201750) [22,23,24,25].

The pivotal role of POR in transferring electrons within the cell begins with the conversion of electrons from nicotinamide adenine dinucleotide phosphate (NADPH) to the co-factor flavin adenine dinucleotide (FAD) [2,26]. Subsequently, these electrons travel from FAD to another flavin co-factor, flavin mononucleotide (FMN). This relay of electrons between FAD and FMN is a key step in the electron transport process. These flavin co-factors, FAD and FMN, are vital components of the POR protein and serve as electron carriers [27]. The electron transfer from FMN further extends to its downstream redox partners, including various cytochrome P450 enzymes [28,29]. POR is vital for many other metabolic processes beyond steroidogenesis and skeletal development. Among these functions are the metabolism of a wide range of substances, including drugs, xenobiotics, arachidonic acid, and eicosanoids, as well as involvement in the synthesis and degradation of vital compounds such as cholesterol, bile acids, and hemoglobin [1,2,12,14].

In the context of drug metabolism, both in vivo and especially in vitro studies consistently underscore the detrimental effects of various POR variants on drug-metabolizing P450 enzymes [14,30,31]. Intriguingly, despite these findings, no clinically relevant adverse drug events have been directly associated with PORD patients to date [32,33]. Nevertheless, a detailed exploration of drug metabolism in a homozygous PORD patient and her heterozygous mother revealed reduced activities in several drug-metabolizing P450 enzymes [11,34,35,36]. Moreover, there is rather poor knowledge about how these variants could affect healthy or subclinical carriers. Thus, what we know about PORD could just be the surface of a complex phenomenon. 

To move toward precise diagnosis and effective management of these complex metabolic disorders, we have designed a workflow. This approach rigorously filters and assesses the pathogenicity of missense variants within the *POR* gene. Furthermore, it advances our comprehension of *POR*-associated disorders and constitutes a valuable contribution to the field of precision medicine. In this regard, we examined a pool of 450 missense variants of the *POR* gene taken from the gnomAD database. Subsequently, we used multiple in silico prediction tools to identify potentially disease-causing variants from this pool. A population analysis and further investigation were conducted on selected variants to validate their pathogenicity. Finally, enzyme kinetics in a model substrate and the activities of major drug-metabolizing CYP450s (CYP3A4, CYP3A5, CYP2C9, and CYP2C19) were assessed with the human POR wild type and selected variants.

## 2. Materials and Methods

### 2.1. Control Variants

The selection of control variants involved an exhaustive review of the existing literature to identify *POR* gene variants with well-documented experimental data [7,10,11,12,13,14,15,16,31]. These control variants were specifically chosen due to their established pathogenic or benign nature, supported by robust experimental evidence. By curating this set of control variants, we ensured a reliable benchmark for evaluating the performance of in silico prediction tools. The selected variants served as a reference group against which the predictions of our chosen in silico tools were compared. This meticulous approach not only bolstered the validity of our study but also enabled a rigorous assessment of the predictive accuracy of the computational tools in distinguishing between pathogenic and benign *POR* gene variants.

### 2.2. Database Collection

The *POR* gene analysis utilized gnomAD V2.1.1 (https://gnomad.broadinstitute.org (accessed on 1 September 2023)), encompassing an extensive dataset of 125,748 exome sequences and 15,708 whole-genome sequences from diverse human populations [37]. This compilation includes individuals from European, Ashkenazi Jewish, East Asian, South Asian, African/African American, Latino/Ad-mixed American, and other ancestry groups. Genomic positions align with the GRCh37/hg19 assembly, while nucleotide positions adhere to the Homo sapiens *POR* mRNA sequence cataloged in the National Center for Biotechnology Information (NCBI) under the reference sequence NM_000941.3. Amino acid positions are denoted in concordance with NCBI’s NP_000932.3 and UniProt’s P16435 numbering systems. In this study, we focused on predicting the pathogenicity of missense variants.

### 2.3. Single Amino Acid Variant Prediction Tools

To assess the potential impact of amino acid substitutions within the *POR* gene, we employed multiple in silico prediction tools. These tools utilize diverse algorithms and databases to predict the functional consequences of genetic variants. Among them are single predictors and meta-predictors; the last ones enhance accuracy by aggregating results from various sources, increasing the reliability of our predictions.

(1) PANTHER

PANTHER (protein analysis through evolutionary relationships) uses evolutionary conservation patterns to predict the functional impact of genetic variants. It is valuable for identifying variants that may disrupt protein structure and function based on evolutionary history [38].

(2) PhD-SNP

PhD-SNP employs support vector machines and sequence-derived features to classify variants as disease-associated or benign. Known for its high accuracy, it is effective in discerning pathogenic mutations from benign ones [39].

(3) SIFT

SIFT (sorting intolerant from tolerant) uses sequence conservation to predict the impact of amino acid substitutions on protein function. It is widely used for identifying deleterious variants that may affect protein function [40].

(4) SNAP

SNAP utilizes neural networks to classify variants based on their potential effect on protein function. It is valuable for distinguishing between tolerated and damaging variants [41].

(5) MAPP

MAPP (multivariate analysis of protein polymorphism) combines sequence information, physicochemical properties, and evolutionary conservation to predict variant pathogenicity. It offers a multifaceted approach to predicting the functional consequences of genetic variants [42].

(6) PolyPhen-1 and PolyPhen-2

PolyPhen (polymorphism phenotyping) evaluates variants by considering sequence-based and structural features. These tools are known for their ability to predict the impact of variants on protein structure and function, with PolyPhen-2 being an improved version [43].

(7) MetaSNP

MetaSNP, a meta-predictor, combines the predictions from multiple existing tools, including PANTHER, PhD-SNP, SIFT, and SNAP, to generate a consensus prediction [44].

(8) MutPred2

MutPred2 utilizes a machine learning approach that considers sequence-based features, structure-based features, and functional annotations to evaluate the pathogenicity of variants. MutPred2 provides a holistic assessment, incorporating diverse data types to enhance prediction accuracy [45].

(9) SNPs&Go

SNPs&Go is a meta-predictor (PHANTHER, PhD-SNP) that integrates protein sequence information with gene ontology annotations to predict the functional impact of variants. This tool provides insights into how variants may disrupt protein function within the context of cellular processes [46].

(10) PredictSNP2

PredictSNP2 employs a machine learning-based approach, considering sequence-derived features, conservation scores, and structural information to make predictions. PredictSNP2′s multi-modal approach (MAPP, PhD-SNP, SIFT, SNAP, PolyPhen-1, and PolyPhen-2) enhances the robustness of predictions, particularly valuable for variants with complex effects [47]. 

### 2.4. Data Integration and Evaluation

The predictions generated by these diverse tools were systematically integrated and analyzed. First, to assess prediction accuracy, we utilized the control variants to compare these results with experimental data from the literature. Based on their performance, we selected tools that discriminated better between pathogenic and nonpathogenic variants to use in the analysis of the variants from the gnomAD database. 

Variants consistently predicted as pathogenic for the selected tools were identified. Accordingly, we selected single-nucleotide variants (SNVs) from the gnomAD dataset that met specific criteria. We chose variants that were consistently predicted as pathogenic by most of the integrated tools [37]. 

In our selection, we focused on variants with low allele frequencies (less than 0.01), indicating their rarity within the population. This approach ensures that the chosen variants are not common background variations. Additionally, we prioritized variants that had not been previously described in the existing scientific literature. 

### 2.5. Expression of POR in E. coli

As a proof of concept, we selected two variants (R268W and L577P) for further investigation to validate our methodology with functional assays in a recombinant system. The cDNAs of the POR wild type (WT), R268W, and L577P, each with a 6xHis tag, were cloned into a pET15b vector and subsequently transformed into *Escherichia coli* BL21 (DE3). Single colonies were selected by growth on LB agar plates containing 100 μg/mL carbenicillin. Large-scale expression was facilitated through an auto-induction system involving the growth of selected colonies in terrific broth with added glucose (0.05%), lactose (0.2%), succinate (20 mM), NaSO_4_ (5 mM), NH_4_Cl (50 mM), MgSO_4_ (2 mM), 0.05 mg/mL riboflavin, and 100 μg/mL carbenicillin. The initial growth occurred at 37 °C until reaching an optical density (OD at 600 nm) of 0.6. Subsequently, the temperature was lowered to 24 °C, and the cultures were grown for an additional 16 h with constant shaking. Bacterial cells were collected by centrifugation, washed with PBS, and suspended in a solution comprising 50 mM potassium phosphate (pH 7.6), 250 mM sucrose, 0.5 mM EDTA, 0.2 mg/mL lysozyme, 1 mM PMSF, and 20 U/mL endonuclease. Spheroplasts were generated through one hour of slow stirring. After centrifugation at 5000× *g* for 20 min, the spheroplasts were suspended in a solution consisting of 50 mM potassium phosphate (pH 7.8), 6 mM MgOAc, 0.1 mM DTT, 20% (*v*/*v*) glycerol, and 1 mM PMSF. Disruption was achieved through sonication, yielding a clear lysate free of cellular debris. Membranes containing the over-expressed POR WT or variants were stored at −80 °C [11,14,15].

### 2.6. Purification of Recombinant Human POR from Isolated E. coli Membranes

All steps were conducted at 4 °C. The His-tagged proteins were solubilized at a concentration of 0.25 g of membrane per mL of 50 mM potassium phosphate (pH 7.4), 10% (*v*/*v*) glycerol, and 1% Triton X-100. The mixture was gently stirred for 16 h and then centrifuged at 12,000× *g* for 15 min. The resulting supernatant was utilized for purification via ion-metal affinity chromatography (IMAC). The supernatant was diluted with buffer A to a final concentration of 50 mM potassium phosphate (pH 7.4), 30 mM imidazole, 0.1% Triton, 150 mM NaCl, and 10% glycerol. This mixture was loaded into 4 mL of His60 Ni SuperflowTM resin, and impurities were washed with buffer A, featuring an increasing concentration of imidazole up to 60 mM. The His-tagged proteins were subsequently eluted with the same buffer, incorporating increasing concentrations of imidazole up to 500 mM. The presence of POR was confirmed through Western blot analysis. Purified samples were concentrated, and the elution buffer was exchanged with 50 mM potassium phosphate (pH 7.4), 10% (*v*/*v*) glycerol, and 0.1% Triton X-100 using Amicon^®^ Ultra centrifugal filters 20,000 MWCO (Sigma-Aldrich, St. Louis, MO, USA). The protein concentration was measured using the Bradford assay [15].

### 2.7. Urea Denaturation Assay

Protein samples were treated with different concentrations of urea up to 4 M to release the flavin molecules from the protein structure. The fluorescence of released FMN and FAD was measured at excitation at 450 nm and emission at 535 nm to determine the flavin content [15,31].

### 2.8. POR Assays with Cytochrome c

The assays were conducted in triplicate in a 96-well format using a Spectramax M2e microplate reader (Molecular Devices, Sunnyvale, CA, USA). Each reaction well consisted of 50 nM POR in a solution containing 50 mM Tris-HCl (pH 7.8) and 150 mM NaCl. The concentration of cytochrome c was varied within the range of 2.5–60 μM. The reactions were initiated by adding 100 µM NADPH and monitored at 550 nm using the extinction coefficient (ε550 = 21.1 mM^−1^ cm^−1^) for a duration of 10 min. The reaction rates were extrapolated from the linear range of the kinetic traces [13,48]. 

The reaction rates for all assays were determined by calculating the slope from the linear range of the kinetic traces. V_max_ and K_m_ values were obtained by fitting the data to the Michaelis–Menten equation and plotted using MATLAB version R2023b (MathWorks, Natick, MA, USA).

### 2.9. Assay of Cytochrome P450 (CYP) Activity in Reconstituted Liposomes

To assess the enzymatic activity of drug-metabolizing cytochrome P450s (CYP3A4, CYP3A5, CYP2C9, and CYP2C19) in the presence of wild-type (WT) or mutant PORs, we employed specific fluorogenic substrates, BOMCC for CYP3A4, CYP3A5, and CYP2C9 and EOMCC for CYP2C19 (Invitrogen Corp, Carlsbad, CA, USA). Purified drug-metabolizing cytochrome P450s (obtained from CYPEX, Dundee, Scotland, UK) were utilized to evaluate the activities of the POR variants, employing 20 μM BOMCC/EOMCC as the substrate.

For in vitro assays, we utilized a reconstituted liposome system consisting of pure WT/mutant POR, the respective CYP450s, and cytochrome b5 in a ratio of 5:1:1. The final assay mixture was composed of 20 µM DLPC (1,2-Dilauroyl-sn-glycero-3-phosphocholine)/DLPGV (1,2-Dilauroyl-sn-glycero-3-phosphoglycerol), proteins (100 nM POR, 20 nM CYP450s, 20 nM b5), 3 mM MgCl_2_, and 20 μM BOMCC in 50 mM Tris-HCl buffer with a pH of 7.4. The reaction volume was set at 100 µL.

To initiate the CYP3A5 reaction, NADPH was added to a final concentration of 1 mM. Subsequently, fluorescence measurements were performed using a Spectramax M2e plate reader (Molecular Devices, Sunnyvale, CA, USA), with excitation at a wavelength of 415 nm and emission at 460 nm for both BOMCC and EOMCC.

### 2.10. Statistical Analysis

For functional assays, three independent experiments were conducted, each in triplicate, for both the POR wild type and mutants. This replication was crucial for ensuring the reliability and reproducibility of our results. To quantify the variability within each set of data, the standard errors (SEs) were calculated. The standard error provides an estimate of how much the sample mean is expected to vary from the true population mean. We established the activity of the POR wild type as 100%, serving as a reference point for calculating the percentage of activity exhibited by the mutants. This approach allowed for a rigorous comparison of the enzymatic activities between the wild-type and mutant POR variants.

Additionally, t-tests were performed to determine the significance of the observed differences between the wild-type and mutant samples. The *p*-value was calculated to assess whether any observed effects were statistically significant. A *p*-value > 0.05 suggests a significant effect on the function of the mutants compared to the wild type. These rigorous statistical approaches enhance the robustness and validity of our findings, allowing us to draw meaningful conclusions from the experimental data.

## 3. Results

### 3.1. Performance of the Prediction Tools

**Figure 1:**
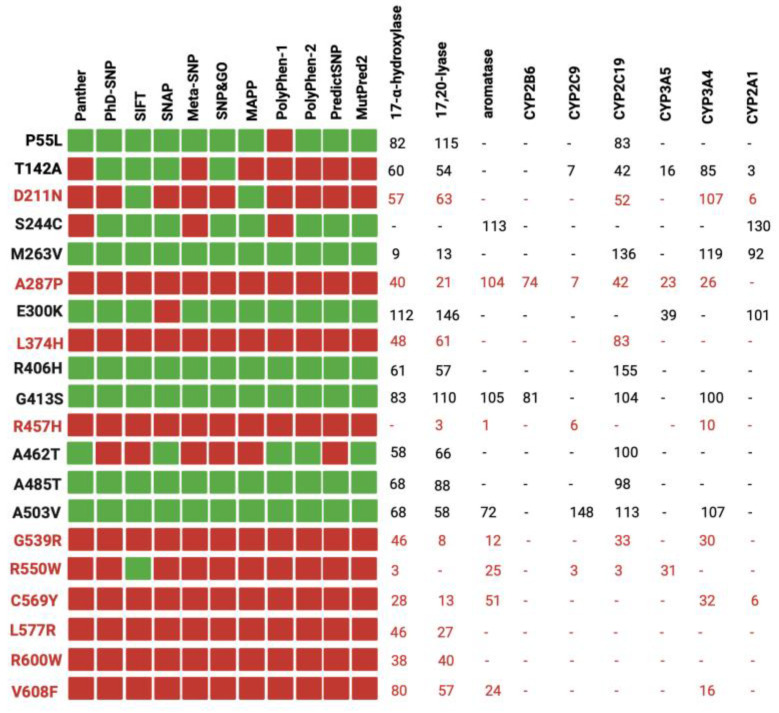
Comparison between in silico tools and experimental data from the literature. To assess the accuracy of the tools, we used known disease-causing and benign variants of human POR as control samples. Our analysis involved comparing the predictions of the tools with the experimental data. In the figure, the prediction of the tools is to be interpreted as red squares for disease-causing variants, while benign variants are denoted by green squares. The variants in red were found in patients with POR deficiency. Created with BioRender.com (accessed on 1 October 2023).

Our study involved a stringent selection process, focusing on 37 reference variants with well-documented experimental data. These data were primarily derived from functional assays measuring the impact of the POR wild type and its mutants on the activities of major steroid and drug-metabolizing CYP450s, as illustrated in Figure 1 [7,29,49,50].

Figure 1 demonstrates a correlation between the computational predictions and the empirical experimental findings. In most cases, variants deemed benign by most of the computational tools (e.g., P55L, R406H, G413S) exhibited over 50% of the redox partner activity. Conversely, variants classified as pathogenic (e.g., R457H, R550W, C569Y) displayed a significant reduction in activity across all redox partners. It is noteworthy that the pathogenic variants were discovered in individuals with severe clinical manifestations, underscoring the robust association between in silico predictions, experimental data, and the clinical phenotypes of carriers [51,52]. However, it is crucial to acknowledge that the impact of the same variant can fluctuate among different redox partners [53,54]. For instance, the case of D211N exhibited over 50% activity with most redox partners but only 6% activity with CYP2A1. A plausible explanation for this observation lies in the distinct interaction of POR with each partner, emphasizing the pivotal role of the specific amino acid alteration and its position within the protein [14,55,56].

**Figure 2:**
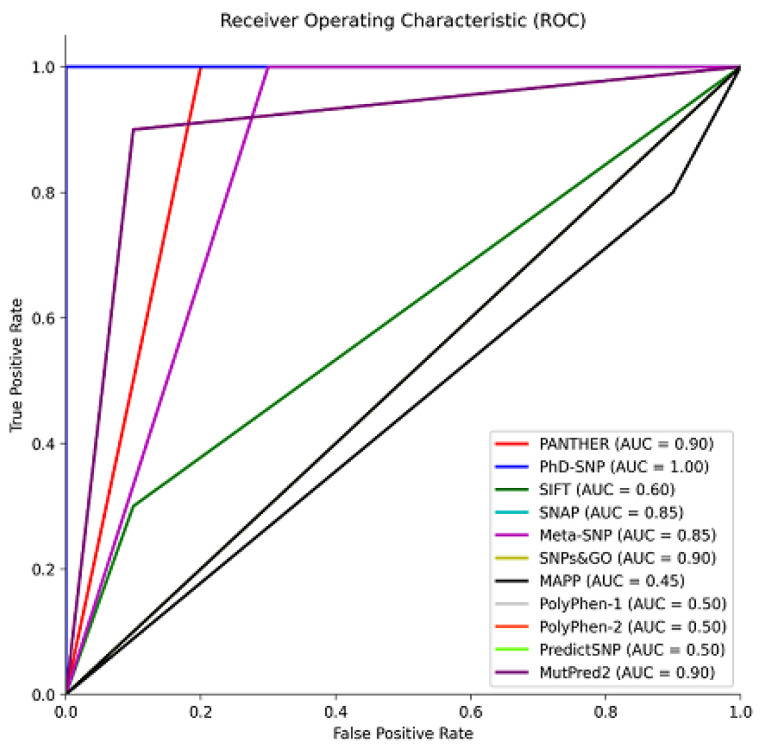
Receiver operating characteristic (ROC) curve illustrating the performance of prediction tools in distinguishing between pathogenic and benign variants. The area under the curve (AUC) values reflect the tools’ accuracy in classifying variants, aiding in the assessment of their pathogenic potential. Data were plotted using MATLAB (MathWorks, Natick, MA, USA).

As shown in Figure 2, to size the predictive performance of these tools, we conducted a receiver operating characteristic (ROC) analysis with the reference group, which allowed us to quantify their ability to distinguish between pathogenic and benign variants. This analysis revealed the tools’ proficiency in accurately classifying variants based on their pathogenic potential. The area under the curve (AUC) is a metric used to assess the performance of classification models. Tools with AUC values ranging from 0.85 to 1 exhibited excellent predictive accuracy, suggesting their effectiveness in accurately classifying variants (Figure 2) [57,58]. On the other hand, SIFT, MAPP, and PolyPhen-1 and -2, with AUC values between 0.45 and 0.6, showed poorer performance and were consequently excluded from the analysis of the missense variants from gnomAD (Figure 2). These results underscore the importance of selecting and utilizing predictive tools with high AUC values for robust variant classification, particularly when evaluating the pathogenic potential of genetic variants in a clinical or research context.

### 3.2. Curating Variants from gnomAD

**Figure 3:**
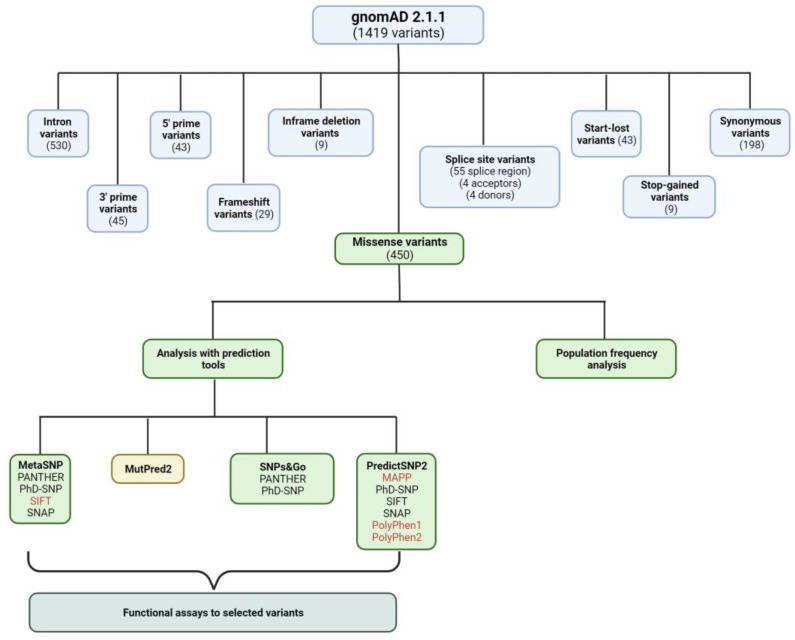
Workflow outlining the curation process of 1419 variants, initially extracted from the gnomAD databases, focusing on the subsequent analysis of 450 missense variants. Notably, in silico tools highlighted in red were excluded from the study as they showed suboptimal performance in the analysis. Created with BioRender.com (accessed on 1 October 2023).

As it is shown in Figure 3, we conducted a comprehensive curation of variants in the POR gene retrieved from the gnomAD databases, which initially comprised a total of 1419 variants. These variants encompassed a diverse range of mutation types, including intron variants, 3′ prime variants, 5′ variants, frameshift variants, in-frame deletions, splice site variants, start-lost variants, stop/gained mutations, synonymous variants, and the predominant category of interest, missense variants [37,59].

Our focus on missense variants was driven by their prevalence, mirroring the clinical scenario in patients with *POR* deficiency, where a significant portion of cases involve carriers with at least one missense variant [2,30,60]. This emphasis is due to the observation that missense variants often retain some residual enzymatic activity, which is critical for sustaining life [7,10]. In contrast, complete loss of POR enzyme activity, which is often the case with deletions or insertions, is incompatible with survival [61,62]. Hence, our decision to prioritize the analysis of missense variants underscores their clinical relevance and potential impact on patient outcomes. We further refined our analysis by applying a series of in silico tools that had previously shown robust performance in our control studies, namely PANTHER, PhD-SNP, SNAP, Meta-SNP, SNPs&GO, and MutPred2. Additionally, allele frequency played a role in our curation process.

### 3.3. Analysis of the Missense Variants

From the pool of 450 missense variants within the *POR* gene, the majority exhibited a minor allele frequency (MAF) of less than 0.01, classifying them as rare variants. Notably, two variants, A503V (MAF 0.3) and T201M (MAF 0.01), stood out as polymorphisms frequently observed in large genome databases. The remaining variants with higher allele frequencies include P228L (MAF 0.003), P181L (MAF 0.002), V472M (MAF 0.002), and V631I (MAF 0.001) [15,37,63]. This distribution highlights the predominantly rare nature of the missense variants within the POR gene, with only a select few displaying higher frequencies, thus underscoring their significance in the context of genetic diversity and disease association.

**Figure 4:**
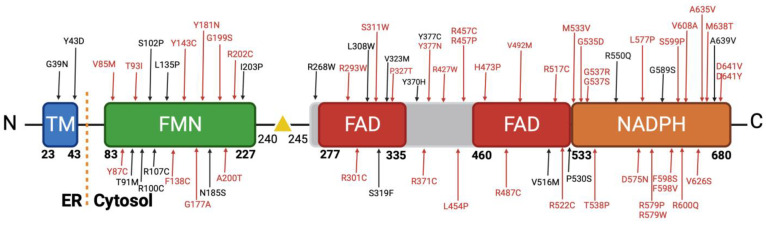
Novel disease-causing variants of human POR. A comprehensive analysis was conducted on approximately 450 missense variants obtained from the Genome Aggregation Database (gnomAD). Utilizing distinct in silico prediction tools, we assessed the potential pathogenicity of these variants. A filtering process was applied to focus exclusively on variants classified as disease-causing. Intriguingly, this rigorous selection yielded 64 variants characterized by remarkably low allele frequencies, with no prior description within the scientific literature. Created with BioRender.com (accessed on 1 October 2023).

Our stringent filtering process led to the identification of 64 novel missense variants (Figure 4). These variants demonstrated consistent classification as pathogenic by our selected in silico tools. As mentioned, they exhibited low allele frequencies, indicating their rarity in the population, which is often associated with more severe clinical phenotypes. Crucially, our literature review confirmed that these 64 variants had not been previously described in patients, further emphasizing their novelty [7,64,65].

These 64 variants are primarily distributed across the co-factor binding domains of the POR protein. Notably, a significant cluster of these variants is situated within the FAD and NADPH binding domains, which have been classified as highly pathogenic (Figure 4). This observation is in concordance with findings in the existing literature, as depicted in Figure 1, where variants within these specific regions consistently exhibit a severe reduction in their activity with redox partners [12,31]. Various factors contribute to the pathogenicity of an amino acid change. Among these factors, the position of the amino acid within the protein is pivotal, with buried amino acids being more likely to induce disease than those located on the protein’s surface. Additionally, the chemical nature of the changes, such as the transition from hydrophobic to hydrophilic amino acids or vice versa, significantly influences their impact on protein function [66].

In our extensive dataset, we identified a significant portion of disease-causing mutations primarily involving arginine (R) and tyrosine (Y) amino acids, contributing 27% and 11%, respectively [66,67]. In the literature, arginine substitutions, including changes to cysteine, proline, and tryptophan, formed a major subset of prevalent disease-causing mutations. We observed variants like R202C, R268W, R293W, R427P, and others (Figure 4). Regarding arginine, its significance lies in its positive charge and participation in important functional and structural roles within proteins [68,69]. Substituting arginine, particularly with amino acids that have distinct chemical characteristics, can disrupt these roles and result in protein malfunction or misfolding, thus contributing to disease. Glycine substitutions to arginine, aspartic acid, glutamic acid, and valine were also common in our dataset, with variants like G39D, G535D, and G37R. As we can see in our dataset with variants G39D, G535D, G37R, and others [70], glycine, being the smallest among the 20 amino acids, is especially sensitive to substitutions with other residues, potentially causing profound alterations to protein structure [66].

Changes in tyrosine residues, as seen in variants like Y43D, Y87C, and Y181N, are harmful due to their involvement in maintaining protein stability, structure, and function. Tyrosine’s chemical properties, including hydrogen bonding capacity and roles in active sites or binding pockets, make it essential. Substituting tyrosine with amino acids of varying properties can disturb the protein’s structure and interactions, potentially leading to functional loss and disease [71,72].

Mutations that change leucine to proline (L454P and L577P; Figure 4) can be detrimental due to the significant differences in their chemical properties. Leucine is a hydrophobic amino acid, while proline has a unique cyclic structure that disrupts protein secondary structures. Substituting leucine with proline can lead to structural distortions, potentially impacting protein function and contributing to disease [66,71].

Of note, our analysis revealed that all 64 pathogenic missense variants were present in a heterozygous state, suggesting that they occur in conjunction with a wild-type copy of the gene or in combined heterozygosity with another variant. This observation raises intriguing possibilities regarding the potential clinical impact of these variants. We postulate that individuals who carry two copies of pathogenic variants, either in a homozygous or combined heterozygous form, may manifest a severe phenotype akin to congenital adrenal hyperplasia (CAH). In less severe cases, these variants could still exert a substantial influence on drug metabolism, potentially resulting in altered drug responses. These findings underscore the importance of further investigations to elucidate the precise clinical ramifications of these newly identified *POR* variants [34,73,74].

### 3.4. Functional Assay of Selected Variants

**Figure 5:**
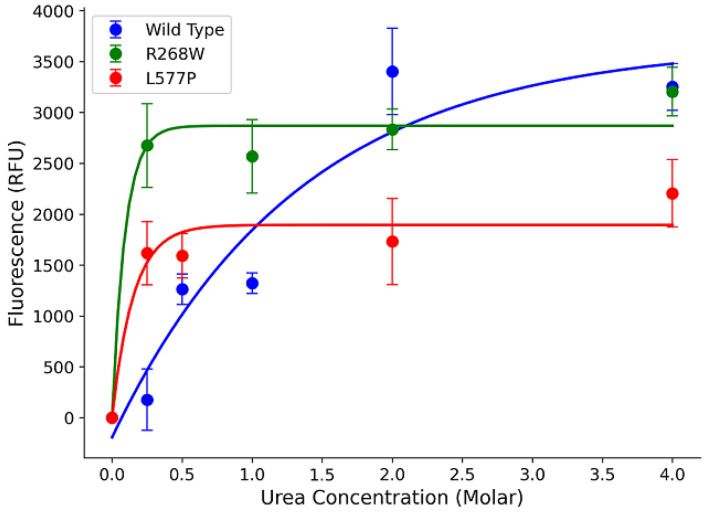
Urea denaturation assay to assess protein stability. Flavin co-factor release was measured through fluorescence, with POR WT showing a gradual release with increasing urea concentration. In contrast, variants R268W and L577P exhibited rapid increases in fluorescence at low urea concentrations, indicating reduced stability. The fluorescence of released FMN and FAD was measured at excitation at 450 nm and emission at 535 nm to determine the flavin content. Data were plotted using MATLAB (MathWorks, Natick, MA, USA).

In our study, we focused on two specific variants, R268W (MAF 0.0002) and L577P (MAF 0.0006), as they were the most frequent among the 64 variants. To assess their impact on POR protein stability, we expressed the wild-type (WT) and variant forms in a bacterial system and performed urea denaturation assays. The fluorescence, which corresponds to flavin co-factor release, served as a proxy for protein structural stability [11,15]. Our results revealed that WT POR steadily released flavins as urea concentration increased, with a peak at 4 M urea (Figure 5). In contrast, the R268W and L577P variants exhibited a rapid increase in fluorescence at a very low urea concentration (0.25 M), which was sustained at higher urea levels. This suggests that these variants are less stable than the WT. Additionally, we observed reduced flavin content in the variants compared to the WT, implying a significant reduction in function (Figure 5). 

**Figure 6:**
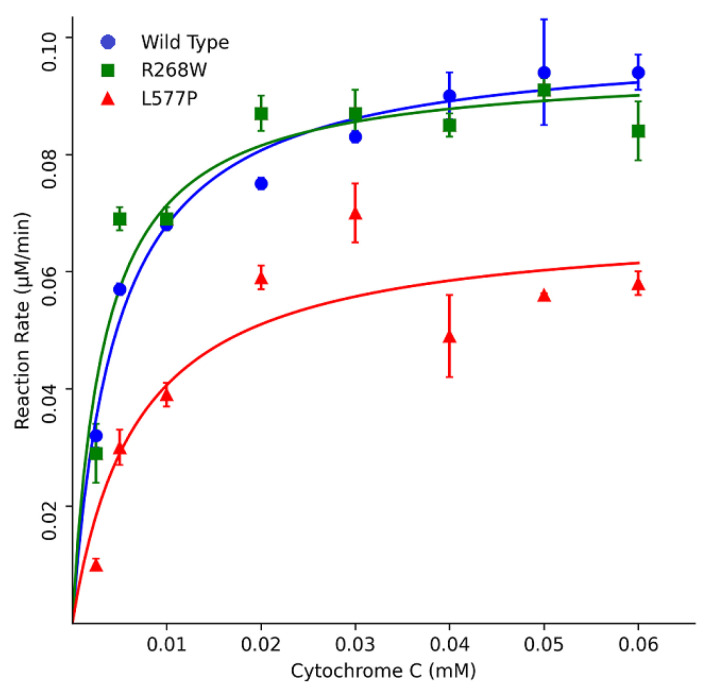
Kinetics assays using cytochrome c as a substrate. POR variants R268W and L577P exhibited varying effects on enzymatic activity compared to WT POR. R268W retained 113% of WT activity, while L577P displayed a more substantial reduction with only 51% activity. Data were plotted using MATLAB (MathWorks, Natick, MA, USA).

Furthermore, kinetic assays using cytochrome c as a substrate indicated that R268W retained 113% of the WT activity, while L577P exhibited a more substantial reduction with only 51% activity. These results were corroborated by different K_m_ values, where L577P showed a higher K_m_ of 6.4 µM and R268W displayed a slightly lower K_m_ of 3.9 µM compared to the WT’s K_m_ of 4.6 µM (Figure 6 and Table 1). The kinetic parameter of POR WT is similar to what was described previously in the literature, a K_m_ around 5 μM [13,14,15]. 

**Figure 7:**
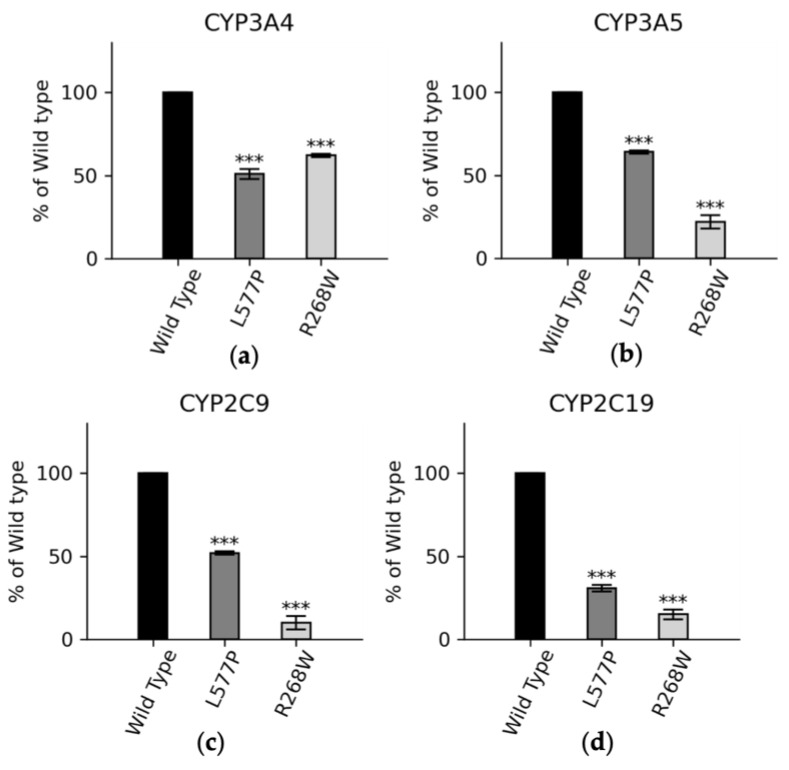
CYP3A4, CYP3A5, CYP2C9, and CYP2C19 activities promoted by WT, L577P, and R268W variants of POR. The activities promoted by the WT POR were set at 100%, and the results are shown as a percentage of WT activity. (**a**) In CYP3A4 assays, POR variants L577P and R268W showed 51% and 62% of WT activity, respectively, while (**b**) in CYP3A5 assays, POR variants L577P and R268W showed 63% and 33% of WT activity, respectively, and (**c**) in CYP2C9 assays, POR variants L577P and R268W showed 52% and 10% of WT activity, respectively. (**d**) In CYP2C9 assays, POR variants L577P and R268W showed 30% and 15% of the WT, respectively. The *p* values can be interpreted as follows: *** (≤0.001).

We also conducted functional assays with major drug-metabolizing CYP450s (Figure 7). In CYP3A4 assays, the POR variants L577P and R268W exhibited 51% and 62% of WT activity, respectively. In CYP3A5 assays, these variants displayed 63% and 33% of WT activity, and in CYP2C9 assays, the variants demonstrated 52% and 10% of WT activity for L577P and R268W, respectively. For CYP2C19, L577P and R268W showed 30% and 15% of WT activity, respectively, highlighting their varying effects on drug metabolism enzymes [14,15,75,76].

## 4. Discussion

A large number of mutations in the *POR* gene have been identified since the early reports describing disordered steroid metabolism linked to bone malformations resembling Antley–Bixler syndrome. Considering the multiple biochemical pathways linked to POR, mutations in POR are likely to have a broad impact on human metabolism. In addition to mutations identified in patients, a large number of genetic variations in the *POR* gene have now been described from genome sequencing studies involving non-clinical populations. However, the metabolic impact or biochemical activities affected by all these variants have not been studied. Here, we have catalogued and studied by computational methods the disease-causing potential of the amino acid variations in POR and tested a couple of variants in a laboratory to test our hypothesis. Our study delves into the intricate landscape of *POR* gene variants, shedding light on the clinical implications and predictive potential of these genetic alterations. The comprehensive evaluation of POR gene variants within the gnomAD database underscores the remarkable rarity of missense variants within this gene [77]. We emphasize that this rarity is a pivotal observation, given the essential role of POR in the function of cytochrome P450 enzymes, which are central players in steroid biosynthesis, drug metabolism, and various physiological processes.

The lack of many known polymorphisms in the *POR* gene is a critical finding. In the broader context of pharmacology and drug metabolism, POR is a backbone in the catalytic cycle of numerous cytochrome P450 enzymes, including CYP1A2, CYP2C9, CYP2C19, CYP2D6, CYP3A4, and CYP3A5, which are vital for the metabolism of a multitude of drugs. In particular, CYP3A4, one of the most abundant CYP450 enzymes in the human liver, is involved in the metabolism of approximately 50% of all marketed drugs [75,78,79]. There is only one major polymorphism in POR, A503V (POR*28, rs1057868), that is present as 15–45% of all alleles across different populations [7]. While most variations in POR tested so far result in reduced enzyme activities of supported enzymes, we have found one variant, Q153R, which showed higher activities of CYP3A4 and CYP3A5 enzymes [14]. Consequently, the scarcity of polymorphisms in the POR gene emphasizes its stability and evolutionary conservation. This knowledge can be harnessed to improve the predictability and safety of drug responses by considering POR genetic variability in drug development and administration [77].

In this study, we analyzed 64 novel pathogenic missense variants within the POR gene, which highlights the clinical relevance of our findings. These variants, primarily clustered within the FAD and NADPH binding domains of the POR protein, have a substantial impact on POR’s ability to transfer electrons to cytochrome P450 enzymes, ultimately affecting drug metabolism [12,80]. We observed that these variants often result in significantly reduced protein stability and function, leading to altered drug responses. Such observations offer insights into the potential clinical consequences for individuals carrying these variants, particularly in relation to drug dosing and efficacy [72]. Notably, our findings highlight the predominance of arginine and tyrosine substitutions among the identified disease-causing mutations, underscoring the clinical significance of these amino acid changes [14,72]. Moreover, our analysis underscores the substantial impact of glycine alterations, emphasizing the importance of specific amino acid characteristics in protein structure and function [81].

Furthermore, our study surpasses mere variant curation. While functional analyses of some variants in POR have been carried out, considering the large number of variants in *POR* derived from genomic studies, the majority of variants in databases have no known significance linked to a potential impact on structure, function, or disease-causing impact. In the current study, we link computational predictions with experimental data, corroborating the pathogenicity of these variants [59]. The strong association we observed between in silico predictions, empirical experimental findings, and clinical phenotypes in patients further validates the effectiveness of our methods. This multifaceted approach can serve as a paradigm for future research in the pharmacogenomics field, where understanding genetic variations and their functional impact on drug-metabolizing enzymes is of paramount importance.

The clinical importance of our findings cannot be overstated [13,82]. As we identify these novel pathogenic variants, it raises the intriguing possibility that individuals carrying two copies of such variants, whether in a homozygous or combined heterozygous form, may manifest severe clinical phenotypes, akin to congenital adrenal hyperplasia (CAH). Since PORD has an autosomal recessive inheritance, two copies of mutations or changes in the *POR* gene are required to cause the disorder, and, therefore, with a gene deletion or truncation in one copy and a severe to medium effect on the other copy, PORD is expected. Even in less severe cases, the presence of such variants can significantly influence drug metabolism, potentially resulting in adverse drug reactions or altered therapeutic outcomes [13,75,78]. Our study underscores the necessity for further investigations into the clinical consequences of these newly identified POR variants, paving the way for precision medicine and personalized drug therapies.

## 5. Conclusions

POR plays a central role in xenobiotic metabolism, and a large number of variations in the *POR* gene are now known due to genome sequencing studies across the world. However, the functional assays or disease-causing potential of these variations in POR are not known. Here, we performed a computational analysis of single amino acid changes in POR for their potential to have a negative impact on the function of POR enzymatic activities and performed a functional analysis on two of the selected variants to test or analyze. The rarity of missense variants in the *POR* gene, the clinical significance of pathogenic variants, and their profound impact on drug metabolism underscore the importance of our study. These findings not only enhance our understanding of *POR* gene variations but also offer a valuable framework for future pharmacogenomic research, with the potential to revolutionize drug development, prescription, and dosing strategies, ultimately improving patient outcomes and drug safety.

## Figures and Tables

**Table 1:** Kinetics parameters using the substrate model cytochrome c.

	Wild Type	R268W	L577P
Vmax (μM/min)	78 ± 3.4	75 ± 1.5	55 ± 3.2
KM (μM)	4.6 ± 0.9	3.9 ± 0.3	6.4 ± 0.5
Vmax/KM	17	19	8.6
%	100	113	51

## Data Availability

Data are available in the manuscript text.
